# α-Crystallin Domains of Five Human Small Heat Shock Proteins (sHsps) Differ in Dimer Stabilities and Ability to Incorporate Themselves into Oligomers of Full-Length sHsps

**DOI:** 10.3390/ijms24021085

**Published:** 2023-01-06

**Authors:** Vladislav M. Shatov, Lydia K. Muranova, Maria A. Zamotina, Nikolai N. Sluchanko, Nikolai B. Gusev

**Affiliations:** 1 Department of Biochemistry, School of Biology, Moscow State University, Moscow 119234, Russia; 2 A.N. Bach Institute of Biochemistry, Federal Research Center “Fundamentals of Biotechnology”, Russian Academy of Sciences, Moscow 119071, Russia

**Keywords:** α-crystallin domain, small heat shock proteins, heterooligomerization

## Abstract

The α-crystallin domain (ACD) is the hallmark of a diverse family of small heat shock proteins (sHsps). We investigated some of the ACD properties of five human sHsps as well as their interactions with different full-length sHsps. According to size-exclusion chromatography, at high concentrations, the ACDs of HspB1 (B1ACD), HspB5 (B5ACD) and HspB6 (B6ACD) formed dimers of different stabilities, which, upon dilution, dissociated to monomers to different degrees. Upon dilution, the B1ACD dimers possessed the highest stabilities, and those of B6ACD had the lowest. In striking contrast, the ACDs of HspB7 (B7ACD) and HspB8 (B8ACD) formed monomers in the same concentration range, which indicated the compromised stabilities of their dimer interfaces. B1ACD, B5ACD and B6ACD transiently interacted with full-length HspB1 and HspB5, which are known to form large oligomers, and modulated their oligomerization behavior. The small oligomers formed by the 3D mutant of HspB1 (mimicking phosphorylation at Ser15, Ser78 and Ser82) effectively interacted with B1ACD, B5ACD and B6ACD, incorporating these α-crystallin domains into their structures. The inherently dimeric full-length HspB6 readily formed heterooligomeric complexes with B1ACD and B5ACD. In sharp contrast to the abovementioned ACDs, B7ACD and B8ACD were unable to interact with full-length HspB1, the 3D mutant of HspB1, HspB5 or HspB6. Thus, their high sequence homology notwithstanding, B7ACD and B8ACD differ from the other three ACDs in their inability to form dimers and interact with the full-length small heat shock proteins. Having conservative primary structures and being apparently similar, the ACDs of the different sHsps differ in terms of their dimer stabilities, which can influence the heterooligomerization preferences of sHsps.

## 1. Introduction

**Figure 1:**
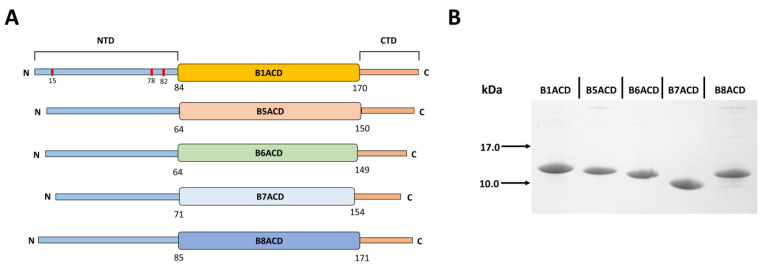
ACDs of selected human sHsps. (**A**) Domain structure of five human sHsps. NTD: N-terminal domain; ACD: α-crystallin domain; CTD: C-terminal domain. Ser residues phosphorylated in HspB1 were marked with red lines and replaced by Asp in 3D mutant. (**B**) Tricine–SDS gel electrophoresis of B1ACD, B5ACD, B6ACD, B7ACD and B8ACD. An amount of ~2 μg of protein was loaded onto each track.

Small heat shock proteins (sHsps) form a large family of ubiquitously expressed proteins. Researchers have detected these proteins in all phyla of life, including viruses, bacteria, plants and animals [1,2,3,4]. sHsps form the first line of defense against different proteotoxic stresses [5]. The monomers of sHsps have a small molecular weight of 12–43 kDa [6,7] as well as a conservative domain structure. The α-crystallin domain (ACD), which consists of 80–100 residues, is a hallmark of the sHsp family. This domain consists of 6–7 β-strands that form an immunoglobulin-like structure that folds into two β-sheets, each of which is formed by 3–4 β-strands [8,9]. This domain plays an important role in the formation of sHsp dimers [8,9,10]. The ACD is the most conservative part of any sHsp [7]. This domain is flanked by rather variable N- and C-terminal domains (Figure 1A). The relatively short C-terminal domain often contains a conservative palindromic sequence: I(V)–X–I(V) [10]. This part of the molecule is highly flexible, and it can either be completely free, remaining unattached, or interact with the hydrophobic groove formed by the β4–β8 strands of the same or neighboring dimer [11,12]. By this means, the C-terminal domain can participate in the formation of small oligomers that contain several sHsp dimers that are “crosslinked” by the I–X–I motif which is hydrophobically patched onto the ACD of the neighboring dimer [13]. The N-terminal domains of sHsps vary in length and structure [7]. This domain seems to play an important role in the formation of large sHsp oligomers that contain 24 or more subunits [6,10,13]. Often, this domain contains phosphorylation sites, and phosphorylation can affect the quaternary structures of sHsps, inducing the dissociation of large oligomers [14,15]. The human genome contains 10 genes that code for sHsps [16,17]. Some sHsps are ubiquitously expressed (HspB1, HspB5, HspB6, HspB8), whereas others (HspB2, HspB3, HspB4, HspB7, HspB9 and HspB10) are tissue-specific [18]. Some sHsps (HspB1, HspB5) form large oligomers that often contain more than 20 subunits, whereas others (HspB6, HspB7, HspB8) tend to form small oligomers (dimers or tetramers) or even monomers [19,20]. Often, several sHsps are simultaneously expressed in the same tissue [18], which favors the formation of heterooligomeric complexes by different members of the sHsp family [20,21,22,23,24]. As already mentioned, the ACD plays a crucial role in the monomer–monomer interaction and has a conservative structure. However, heterooligomerization mechanisms and the role of the ACD in these processes remain poorly understood.

Therefore, in this work, we aimed to analyze the structure and properties of the ACDs of five widely expressed sHsps to assess their abilities to interact with different full-length sHsps and incorporate themselves into their oligomeric structures.

## 2. Results

### 2.1. Oligomeric States of ACDs of Different Small Heat Shock Proteins

By using ion-exchange and size-exclusion chromatography, we obtained highly pure ACDs of five human small heat shock proteins (Figure 1A), with an apparent molecular weight on the SDS gel electrophoresis in the range of 9–12 kDa (Figure 1B).

**Figure 2:**
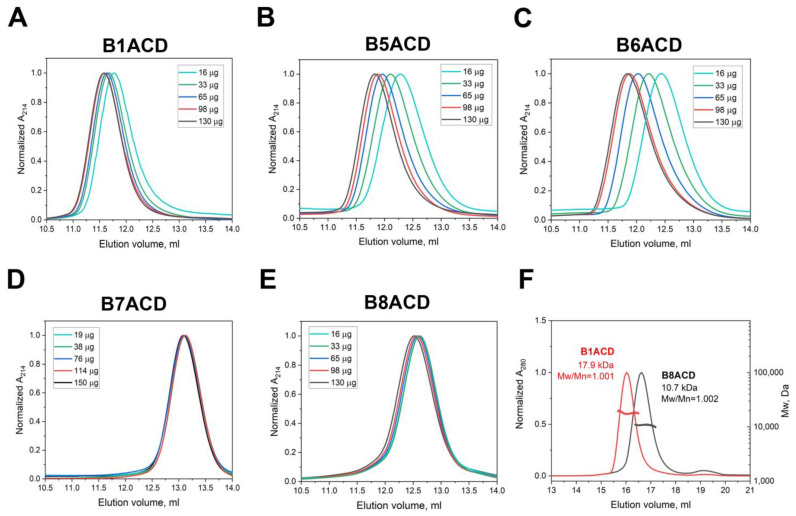
The five sHsp ACDs had markedly different dimer stabilities. (**A**–**E**) Size-exclusion chromatography of different ACD quantities loaded on a Superdex 75 10/300 column. The quantity of protein loaded on the column is color-coded for each panel. Representative results of no less than three experiments are presented. (**F**) SEC–MALS-derived absolute Mw determination for two contrasting species, B1ACD and B8ACD, run on a Superdex 200 Increase 10/300 column at identical protein load concentrations (2 mg/mL) and volumes (90 µL). Mw distributions are indicated across peaks along with corresponding polydispersity indices (Mw/Mn).

All the ACDs eluted as symmetrical peaks on a Superdex 75 10/300 column (Figure 2). The elution volumes of B7ACD and B8ACD were independent of the quantity of protein loaded on the column. The molecular weight of B8ACD, determined by SEC–MALS, was equal to 10.7 kDa, which is close to 9.6 kDa, i.e., the calculated molecular weight of B8ACD monomers. Due to the low extinction coefficient of B7ACD, we were unable to reliably determine its molecular weight via SEC–MALS. However, because the relatively large elution volumes of both B7ACD and B8ACD were independent of the protein quantity loaded on the column, we concluded that the ACDs of these two proteins were present as monomers.

Three other ACDs (B1ACD, B5ACD, B6ACD) also eluted as symmetrical peaks; however, the elution volumes of these domains were strongly dependent on the quantity of protein loaded on the column (Figure 2A–C). For instance, the increase in the B1ACD quantity loaded on the column from 16 μg to 130 μg was accompanied by a decrease in the elution volume from 11.95 to 11.60 mL. The absolute Mw of the B1ACD at a 180 μg loading determined from the SEC–MALS was equal to 17.9 kDa, which corresponded to the dimeric state of this protein (calculated monomer Mw = 9.7 kDa) (Figure 2F). We also observed qualitatively similar but much more pronounced concentration-dependent chromatographic peak shifts for B5ACD and B6ACD. The dependence of the elution volume on the protein concentration indicated that these domains were present in the form of an equilibrium mixture of monomers and dimers.

According to the presented data, the ACDs of HspB1, HspB5 and HspB6 form dimers at high protein concentrations, whereas the dilution results in their dissociation from dimers to monomers. We plotted the elution volume against the concentration of ACD loaded on the column and used the hyperbolic approximation V_e_ = A − B*c/(K + c), where *V_e_* is the elution volume, *A* and *B* are constants, *K* is the dissociation constant, and *c* is the ACD concentration. By using this approximation, we determined the apparent dissociation constants for the B1ACD, B5ACD and B6ACD dimers as 27, 127 and 162 µM, respectively. Thus, the HspB1, HspB5 and HspB6 ACDs were present as a monomer/dimer mixture. B1ACD formed the most stable dimers, withstanding the substantial dilution, whereas the HspB7 and HspB8 ACDs were predominantly present as monomers.

**Figure 3:**
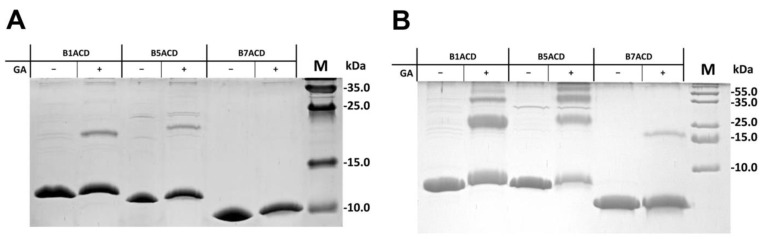
Chemical crosslinking of B1ACD, B5ACD and B7ACD by glutaraldehyde (GA). We crosslinked all ACDs at equal concentrations (3 mg/mL) by (**A**) 0.008% or (**B**) 0.024% glutaraldehyde and subjected them to Tricine–SDS–PAGE. Representative results of no less than five experiments are presented.

We further used chemical crosslinking and dynamic light scattering (DLS) for the independent confirmation of this conclusion. For the chemical crosslinking by glutaraldehyde, we chose B1ACD, B5ACD and B7ACD because only these ACDs contained equal numbers of Lys residues in their putative dimeric interfaces involving the antiparallel β6+7 strands that would provide for the most correct comparison. At a relatively high protein concentration (3 mg/mL), the incubation with low concentrations of glutaraldehyde induced the formation of the crosslinked dimers of B1ACD and B5ACD, while B7ACD remained completely monomeric (Figure 3A). At a high glutaraldehyde concentration, large portions of B1ACD and B5ACD became crosslinked, whereas B7ACD remained predominantly uncrosslinked (Figure 3B). We obtained similar results with DLS. Indeed, at a high protein concentration (3 mg/mL), the diameters of the B1ACD and B5ACD particles were equal to 4.39 ± 0.29 and 4.34 ± 0.28 nm, respectively, whereas the diameter of B7ACD was equal to 3.18 ± 0.31 nm. Assuming that all the ACDs had similar globular structures and using the earlier described approach [25], we were able to estimate the molecular weights of the B1ACD and B5ACD as 16.2 and 15.9 kDa, respectively, whereas that of the B7ACD was equal to 9.4 kDa. Collectively, our data indicate that B1ACD and B5ACD predominantly formed dimers, whereas B7ACD remained in the monomeric form.

### 2.2. Interaction of ACDs with Small Heat Shock Proteins Forming Large Oligomers

The ACD plays an important role in the dimerization of sHsps [9,26] and the stabilization of the large oligomers formed by certain sHsp members, such as HspB1 and HspB5. Therefore, it was reasonable to analyze the interaction of the selected ACDs with full-length sHsps that form different-sized oligomers.

**Figure 4:**
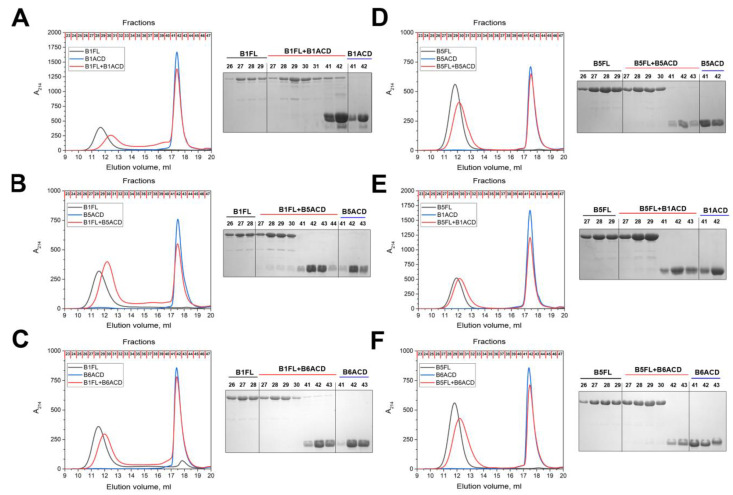
Interaction of selected ACDs with large oligomers of (**A**–**C**) HspB1 and (**D**–**F**) HspB5. Elution profiles of isolated full-length (FL) small heat shock proteins (black lines), isolated ACDs (blue lines) and their mixtures (red lines) on a Superdex 200 Increase 30/100 column. The protein composition of the fractions collected during each elution profile (no less than three runs) and analyzed by SDS–PAGE on the right-hand panel of each elution profile are presented.

Using size-exclusion chromatography, we analyzed the interaction of the ACDs with the large oligomers formed by full-length HspB1 and HspB5 (Figure 4). Due to the different molecular weights, the peaks of the full-length sHsps and peptides are well separated and do not overlap with each other (Figure 4). The addition of B1ACD, B5ACD or B6ACD to the large oligomers of HspB1 (Figure 4A–C) and HspB5 (Figure 4D–F) was not accompanied by the effective incorporation of the ACDs into the oligomers formed by the full-length sHsps, according to the SDS–PAGE analysis of the fractions obtained during the SEC. However, the addition of the α-crystallin domains was accompanied by the shifting of the peak that corresponded to the full-length proteins to larger elution volumes, as well as its broadening (Figure 4). This indicates that the ACDs transiently interacted with the large oligomers formed by the full-length sHsps, which changed their hydrodynamic properties and led to their retardation on the column. The rather weak interaction of the ACDs with the large sHsp oligomers was apparently not accompanied by the efficient replacement of the monomers of the corresponding full-length proteins by the corresponding ACDs or by the incorporation of the ACDs into the structures of the large oligomers. In the case of the mixture of B5ACD and full-length HspB1 (Figure 4B), we detected small quantities of the ACD peptide in the trailing edge of the peak formed by the large oligomers of the full-length protein; in the case of B1ACD and full-length HspB1, we also detected small quantities of full-length HspB1 in the peak that corresponded to B1ACD (Figure 4A).

**Figure 5:**
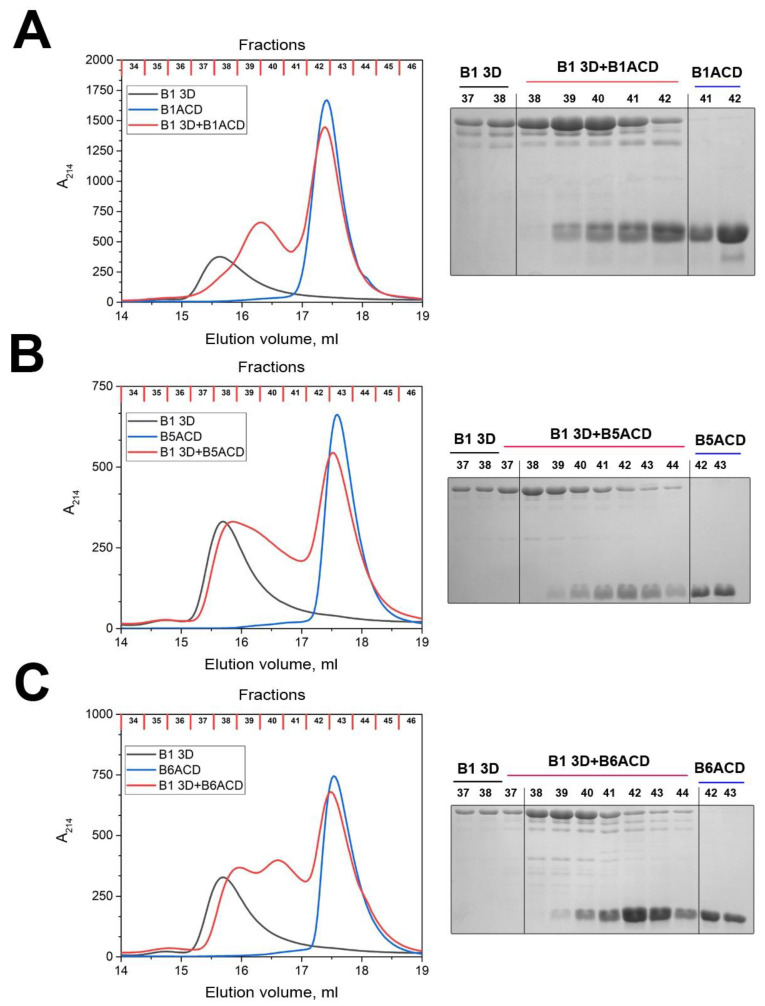
Interactions of (**A**) B1ACD, (**B**) B5ACD and (**C**) B6ACD with a 3D mutant of HspB1. Elution profiles of the isolated 3D mutant (black lines), isolated ACDs (blue lines) and their equimolar mixture (red lines) on a Superdex 200 Increase 10/300 column. The protein compositions of fractions collected during each chromatography run (no less than three runs) and determined by SDS–PAGE are presented on the right.

Nevertheless, the presented data indicate that B1ACD, B5ACD and B6ACD only weakly interacted with the large oligomers formed by full-length HspB1 or HspB5, which was likely due to their inability to efficiently outcompete the multiple chemical contacts that full-length HspB1 or HspB5 protomers establish within their oligomers using not only their ACDs, but also their N- and C-terminal domains. To provide further insight, we analyzed the interaction of the abovementioned ACDs with the so-called 3D mutant of HspB1. This protein contains three point mutations, S15D, S78D and S82D, which mimic the phosphorylation of this protein at the indicated Ser residues located within the unstructured N-terminal region (Figure 1A). Phosphomimicking mutations induce the partial dissociation of large HspB1 oligomers and the accumulation of smaller species (probably dimers or tetramers) [27,28]. The 3D mutant of HspB1, with an apparent molecular weight of 100–120 kDa, was well separated from the analyzed ACDs (Figure 5). When the mixtures of the 3D mutant and ACDs were loaded on the column, we detected new protein peaks with intermediary elution volumes between those of the individual 3D mutant and individual ACDs (Figure 5). The fractions of these peaks contained both the 3D mutant and ACDs (Figure 5). Moreover, in the peak that corresponded to the individual ACDs (Fractions 41–43), we detected monomers of the full-length 3D mutant (Figure 5). These results strongly indicate that B1ACD, B5ACD and B6ACD are readily incorporated into the structures of the oligomers formed by the 3D mutant of HspB1, and that the ACD incorporation is accompanied by the displacement of the 3D mutant subunits.

**Figure 6:**
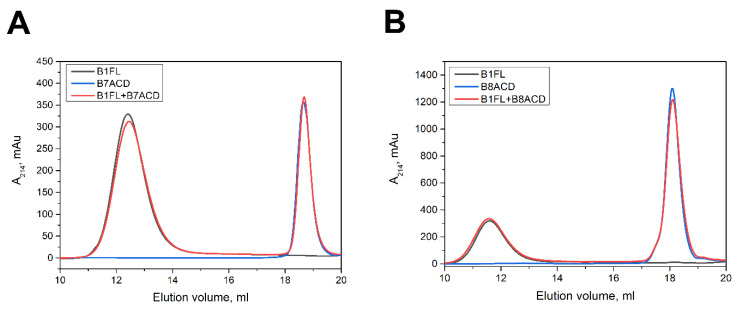
Interactions of (**A**) B7ACD and (**B**) B8ACD with HspB1. Elution profiles of full-length (FL) HspB1 (black lines), B7ACD and B8ACD (blue lines), and an equimolar mixture of HspB1 and the corresponding α-crystallin domains (red line) on a Superdex 200 Increase 10/300 column.

Both HspB8 and HspB7 are expressed in the cardiac and skeletal muscles [18,29], which also express HspB1, HspB5 and HspB6. Therefore, as part of full-length proteins, the ACDs of HspB7 and HspB8 can, in theory, interact with their paralogs. By using size-exclusion chromatography, we examined the interaction of B7ACD and B8ACD with the full-length HspB1 (Figure 6). In neither case were we able to detect any substantial changes in the elution profiles that could be interpreted as protein–protein interactions. We obtained similar results in the experiments in which we analyzed the interaction of B7ACD and B8ACD with full-length HspB5 (Supplementary Material, Figure S1). Thus, in contrast to B1ACD, B5ACD and B6ACD, the crystallin domains of HspB7 and HspB8 were unable to form even transient complexes with HspB1 or HspB5. As already mentioned, B1ACD, B5ACD and B6ACD more effectively interacted with the 3D mutant of HspB1, forming smaller oligomers than the large oligomers formed by wild-type HspB1 (see Figure 5). Therefore, we next analyzed the interaction of B7ACD or B8ACD with the 3D mutant of HspB1 (Supplementary Material, Figure S2). However, using size-exclusion chromatography, we were again unable to detect any signs of interaction. Thus, in contrast to B1ACD, B5ACD and B6ACD, the crystallin domains of HspB7 and HspB8 cannot stably interact and are not incorporated into the oligomers formed by HspB1 (or its 3D mutant) or HspB5.

### 2.3. Interaction of ACDs with Smaller sHsp Oligomers

**Figure 7:**
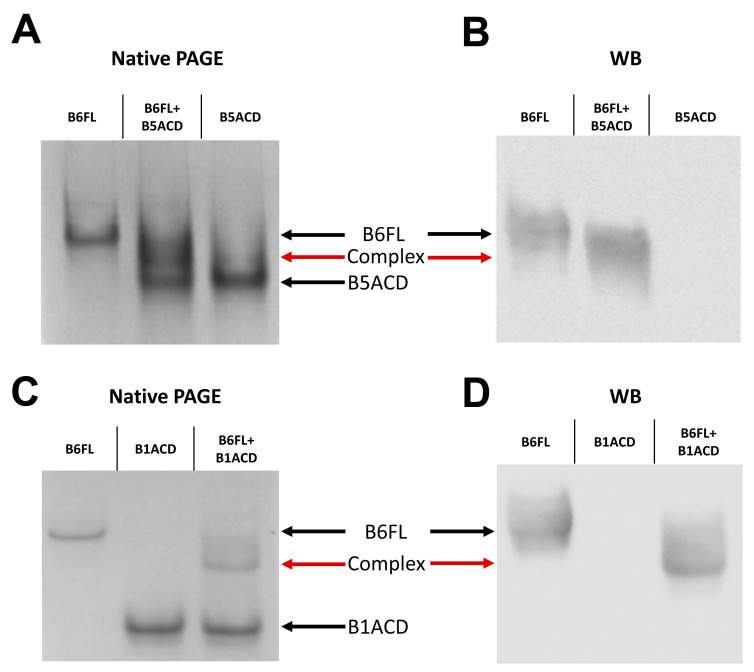
Interaction of full-length HspB6 (B6FL) with (**A**,**B**) B5ACD and (**C**,**D**) B1ACD, analyzed by (**A**,**C**) native PAGE, followed by (**B**,**D**) Western blotting stained with monoclonal anti-HspB6 antibodies. The positions of the HspB6, ACDs and their complexes are marked with arrows. Representative results of no less than five experiments are presented.

The full-length HspB6, HspB7 and HspB8 form relatively small oligomers that are composed of monomers or dimers [9,20,30,31], the typical molecular weights of which only slightly differ from those of the selected ACDs. Therefore, size-exclusion chromatography cannot provide the effective separation of these proteins. To overcome this problem, we used clear (stainless) native gel electrophoresis that we ran in 80 mM of glycine–Tris (pH 8.6) [32]. Under these conditions, electrophoretic mobility depends on the size, form and charge of the analyzed proteins, and hence, it provides for the necessary resolving power. Indeed, full-length HspB6 possessed low electrophoretic mobility, whereas the electrophoretic mobilities of B5ACD, and especially B1ACD, were higher (Figure 7).

**Figure 8:**
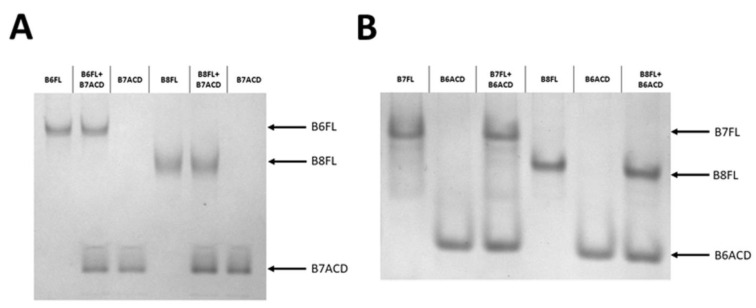
(**A**) Interaction of full-length HspB6 (B6FL) and HspB8 (B8FL) with B7ACD, analyzed by native PAGE. (**B**) Interactions of full-length HspB7 (B7FL) and HspB8 (B8FL) with B6ACD. The positions of the full-length proteins and α-crystallin domains are marked with arrows.

When we mixed full-length HspB6 with B5ACD or B1ACD, the band that corresponded to full-length HspB6 vanished and new bands that possessed intermediate electrophoretic mobilities appeared on the gel (Figure 7). We confirmed the formation of the heterooligomeric complex of full-length HspB6 with the corresponding peptides by immunoblot staining with monoclonal anti-HspB6 antibodies (Figure 7B,D). According to the results, B1ACD and B5ACD interacted and formed complexes with full-length HspB6. We used the same approach for analyzing the interactions of HspB6 and HspB8 with B7ACD (Figure 8A), as well as the interactions of full-length HspB7 and HspB8 with B6ACD (Figure 8B). In a similar experiment, we also analyzed the interactions of HspB6 and HspB7 with B8ACD (Supplementary Material, Figure S3). However, in no case did we detect the interaction of the analyzed ACDs with full-length HspB6, HspB7 or HspB8.

## 3. Discussion

The ACDs of the five human sHsps have similar sizes (about 85 residues) and according to the PsiPred analysis [33], they have practically identical secondary structures (data not presented). According to previous investigations, at high concentrations, ACDs tend to form stable dimers [8,9]. However, in this study, we demonstrated that the dilution of some ACDs (namely, B1ACD, B5ACD and B6ACD) provoked dimer dissociation, which indicated the substantial differences in the stabilities of the ACD dimeric interfaces (Figure 2). In striking contrast, the dilution of the other two ACDs, B7ACD and B8ACD, had no effect on the oligomeric state of these proteins, which remained mostly monomeric in a wide range of protein concentrations (Figure 2).

**Figure 9:**
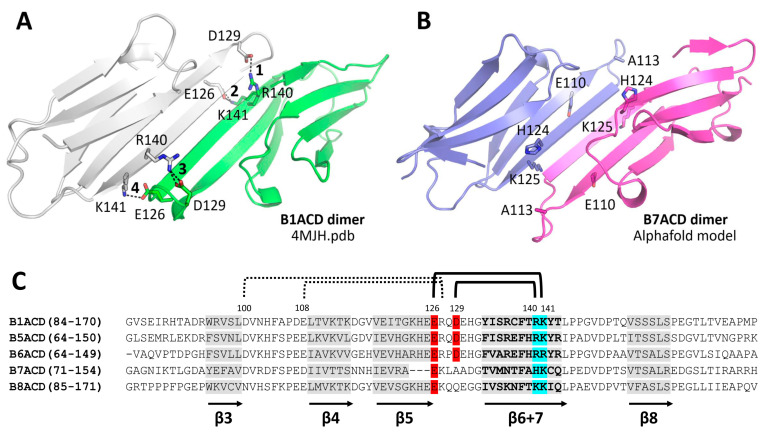
Structural determinants of the different stabilities of the dimeric interfaces of sHsp ACDs. Structural comparison of (**A**) B1ACD and (**B**) B7ACD indicating differences in the numbers of intermolecular salt bridges. The four bridges found in the B1ACD structure are numbered 1–4 and key residues involved in the formation of salt bridges in B1ACD, or equivalent residues in B7ACD, are labeled. (**C**) Alignment of α-crystallin domains of five representative human small heat shock proteins. β-strands are marked in grey and highlighted by arrows. Negatively charged residues that form salt bridges are marked in red and positively charged residues involved in salt bridge formation are marked in blue. Main salt bridges between monomers are indicated by solid brackets and auxiliary contacts are indicated by dashed brackets. Numbering above alignment corresponds to HspB1. For multiple alignments generated by T-Coffee [35] (Available online: http://tcoffee.org accessed on 15 September 2022), we used the following sequences from UniProt: P04792 (HspB1), P02511 (HspB5), O14558 (HspB6), Q9UBY9 (HspB7) and Q9UJY1 (HspB8).

The question arises as to why ACDs derived from different sHsps and with similar primary and secondary structures differ in their quaternary structures. Within the canonical ACD dimer, the subunit interface is formed by long antiparallel β6–β7 strands that are stabilized by the backbone hydrogen bonds and salt bridges between the side chains of the semiconserved residues (Figure 9) [10,34]. 

For instance, the B1ACD interface is stabilized by ten H-bonds that are formed by the backbone CO and NH atoms, as well as by four salt bridges formed by Arg140–Asp129 and Lys141–Glu126 (Figure 9A). Additional salt bridges can be formed between Arg127 and Glu108 of two neighboring subunits and between Arg127 and Asp100 within the same subunit in the vicinity of the dimer interface [9,34,36]. All these salt bridges are preserved in B1ACD, B5ACD and B6ACD, which all maintain the structure of the canonical ACD dimer (Supplementary Material, Figure S4). The difference in the apparent dissociation constants of these ACDs can be caused by the difference in the number of hydrogen bonds that are formed between their subunits. Indeed, the PRODIGY algorithm [37], which takes into account the contribution of hydrogen bonds, predicted the K_d_ values for B1ACD, B5ACD and B6ACD as 50, 150 and 530 nM, respectively. Although likely somewhat overestimated in terms of their absolute values, these affinities perfectly correlate with the differences in the dimer stabilities that we observed in our SEC experiments (Figure 2). In the case of B7ACD and B8ACD, the dimeric structures of which have not been reported yet but can be reliably modeled using AlphaFold 2 [38] (Figure 9B and Supplementary Material, Figure S4), the conservative Arg residue homologous to the Arg140 of HspB1 is replaced by His (HspB7) or Lys (HspB8), the conservative Arg residue homologous to the Arg127 of HspB1 is replaced by Lys and the conservative Asp homologous to Asp129 of HspB1 is replaced by either Ala (HspB7) or Gln (HspB8) (see Figure 9C). In contrast to B1ACD, B5ACD and B6ACD, which form the canonical ACD dimers that are easily reconstructed by Alpha Fold 2, B8ACD and especially B7ACD dimers cannot be as unequivocally reconstructed (Supplementary Material, Figure S4). Indeed, while five independent predictions for B1ACD, B5ACD and B6ACD resulted in ACD dimers with excellent reproducibility (Ca RMSD << 1 Å), the ACD dimers for B8ACD were much less consistent (Ca RMSD >> 1Å) (Supplementary Material, Figure S5). The ACD dimers for B7ACD were extremely inconsistent and the canonical ACD interface was preserved only seldomly in the models (Supplementary Material, Figures S4 and S5). According to the exemplary canonical-like model of the B7ACD dimer (Figure 9B), there was a much shorter β6–β7 strand, conferring only 6 H-bonds for the stabilization of the hypothetical interface (compared with the 10 H-bonds in the case of B1ACD), and a complete lack of stabilizing salt bridges. This analysis provides relevant structural insights into the observed differences in the ACD dimer stabilities for the studied sHsp paralogs.

As already mentioned, the ACD plays an important role in intermonomer interaction, and it therefore affects the formation and overall organization of sHsp oligomers. Earlier, researchers demonstrated that B5ACD can interact and affect the oligomeric structures of large full-length HspB5 oligomers [13]. We confirmed these data and demonstrated that B1ACD, B5ACD and B6ACD transiently interact with the large oligomers of full-length HspB1 and HspB5 (Figure 4). The addition of these ACDs was accompanied by the shifting of HspB1 and HspB5 on the elution profile and the partial broadening of the peak that corresponded to the full-length proteins. However, we only detected minor quantities of the ACD peptides in the peak of the full-length proteins, which means that these peptides only transiently interact with the large oligomers of HspB1 and HspB5. We can perhaps explain the low efficiency of the interaction by the multipoint contacts that stabilize each sHsp protomer within the large oligomers (often containing more than twenty subunits). We therefore hypothesized that the efficiency of the peptide interaction can be increased by decreasing the number of subunits in the oligomers of full-length sHsps. To evaluate this assumption, we used the 3D mutant of HspB1, in which we replaced the three Ser residues (Ser15, Ser78 and Ser82 (Figure 1A)) with Asp, thus mimicking the phosphorylation of these sites [14]. The 3D mutant forms small oligomers that are composed of the dimers or tetramers of HspB1 [27,28,30]. B1ACD, B5ACD and B6ACD effectively interacted with the 3D mutant of HspB1 and formed heterooligomers with this protein (Figure 5). We were unable to follow all the steps of the heterooligomer formation. However, according to the published data [13], in the beginning, the individual ACD species are anchored by the free C-terminal extensions of the monomers that belong to the large oligomers. Subsequently, the α-crystallin domains of the incoming peptides and α-crystallin domains of the already assembled subunits interact with each other, and this process is accompanied by a (partial) displacement of the previously assembled monomers and the incorporation of the incoming ACDs into the heterooligomer.

We also examined the interaction of B1ACD and B5ACD with full-length HspB6 (Figure 7). Full-length HspB6 is predominantly present in the form of dimers [19], thus making the interaction of ACDs with it more probable. Indeed, by using native gel electrophoresis, we detected the formation of a new band that corresponded to heterooligomers formed by B1ACD and B5ACD with full-length HspB6 (Figure 7).

Finally, we analyzed the interactions of B6ACD, B7ACD and B8ACD with different sHsps. B7ACD and B8ACD are predominantly present as monomers, whereas B6ACD can form dimers. We expected that the monomers would more easily interact and insert themselves into the structures of the sHsp oligomers. However, neither B7ACD nor B8ACD was able to interact with the large oligomers of HspB1 or HspB5 (Figure 6). We performed similar experiments with the 3D mutant of HspB1 (Supplementary Material, Figure S2). Even in this case, we were unable to detect any interactions of these ACDs with the full-length mutant of HspB1 (Supplementary Material, Figure S2). We failed to detect the interaction of B7ACD with full-length HspB6 or HspB8 (Figure 8A), the interaction of B8ACD with full-length HspB6 or HspB7 (Supplementary Material, Figure S3) or an interaction between full-length HspB7 and HspB8 and B6ACD (Figure 8B). Researchers have previously reported the interaction of full-length HspB6 and HspB7, as well as the interaction of full-length HspB7 with HspB8 [20,39]. We can explain this apparent discrepancy by the much lower affinities of short isolated ACDs with their partners than those of full-length proteins with theirs.

**Figure 10:**
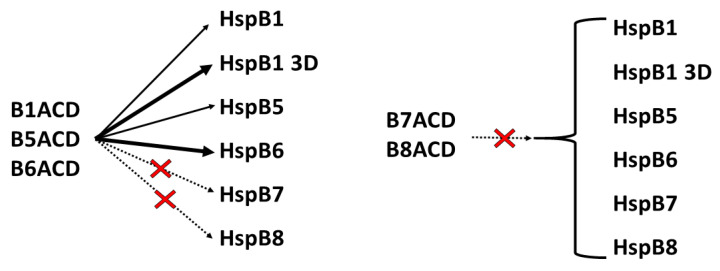
Heterooligomerization scheme of ACDs and full-length sHsps. Thick black lines represent strong interactions, thin black lines represent weak interactions and dashed black lines with red crosses represent a lack of interaction.

We present the main results of the study in the scheme in Figure 10. B1ACD interacted with full-length HspB1, its 3D mutant, HspB5 and HspB6; however, it failed to interact with full-length HspB7 or HspB8. Similar behavior was characteristic for B5ACD and B6ACD. These three ACDs have similar primary structures and properly located charged residues and hydrogen bonds that provide the high stabilization of the dimers formed by these peptides. These ACDs more effectively interact with small oligomers (3D mutant of HspB1, full-length HspB6) than with large oligomers of HspB1 and HspB5. B7ACD and B8ACD also have a conservative structure that is characteristic of the α-crystallin domain. However, their charged residue distributions are less favorable than those of the three abovementioned ACDs, and therefore, B7ACD and B8ACD were present in monomeric forms. The predicted structure of the ACD dimers of HspB8, and especially of HspB7, was different from that of the canonical dimeric ACDs of HspB1, HspB5 and HspB6 (Figure 9 and Supplementary Material, Figure S4). Therefore, B7ACD and B8ACD cannot productively compete with the α-crystallin domains of HspB1, HspB5 or HspB6, and they cannot displace the monomers of these proteins. Full-length HspB7 and HspB8 predominantly form monomers [20,40], and hence, they do not interact with B1ACD, B5ACD or B6ACD.

The ACDs of all sHsps are the most conservative parts of their molecules as well as the hallmark of the sHsp family [7]. Therefore, it was supposed that the ACD does not play a crucial role in sHsp heterooligomerization and that this process is predominantly determined by the highly variable N-terminal domain and/or C-terminal extension that contains the hydrophobic I–X–I tripeptide [13,24,41]. According to our results, while they have similar primary and predicted secondary structures, the ACDs of the different small heat shock proteins differ in terms of their dimer stabilities. This difference is correlated with the distribution of the charged residues and H-bonds in the monomer/monomer interface, and it can potently influence both the homo- and heterooligomerization of sHsps. Thus, the formation of heterooligomers depends not only on the structure and properties of the N-terminal domain or C-terminal extension, but also on the minor variations in the ACD structure.

## 4. Materials and Methods

### 4.1. Expression and Purification of ACDs

We used pET23b constructs that contained the full sequence of either human HspB1, HspB5 (also called αB-crystallin), HspB6, HspB7 or HspB8 [42] for obtaining the fragments that corresponded to the α-crystallin domain (ACD). The forward and reverse primers used in the PCR are presented in the Supplementary Material, Table S1. We digested the PCR products with *NdeI* and *XhoI* and cloned them into pET23b, which we similarly digested. The integrity of all the expression constructs and the lack of mutations were confirmed by DNA sequencing. We performed the expressions of all the ACDs in *E. coli*, as described earlier [9]. The recombinant fragments contained residues 84–170 of HspB1, residues 64–150 of HspB5, residues 64–149 of HspB6, residues 71–154 of HspB7 and residues 85–171 of HspB8 (Figure 1A). In other words, they lacked the variable N- and C-terminal fragments of the analyzed proteins and contained the complete sequence of the ACD, including the β2–β8 strands of each sHsp. We performed the expressions of the full-length recombinant sHsps as described earlier [20,30].

We purified the recombinant ACDs with a combination of ammonium sulfate fractionation (20–60% saturation), ion-exchange chromatography (High Trap Q) and size-exclusion chromatography (Superdex 200 26/600 pg). We dialyzed the purified ACDs against buffer B (20 mM Tris–acetate, pH: 7.6, 10 mM NaCl, 0.1 mM PMSF, 2 mM DTT), evaluated their purity using Tricine–SDS–PAGE [43] (Figure 1B) and stored them at −20 °C. We spectrophotometrically determined protein concentration using an A_280_^0.1%^ equal to 0.87 for B1ACD, 0.15 for B5ACD, 0.16 for B6ACD and B7ACD and 0.73 for B8ACD. The other physicochemical properties of the different ACDs are presented in the Supplementary Material, Table S2.

The full-length sHsps were purified with a combination of ammonium sulfate fractionation, ion-exchange or hydrophobic chromatography and size-exclusion chromatography, as described earlier [20,30]. We dialyzed all the proteins against buffer B and stored them at either −20 or −80 °C. Protein concentration was determined either by the absorbance at 280 nm using A_280_^0.1%^ equal to 1.78 for HspB1, 0.69 for HspB5, 0.58 for HspB6 and 1.23 for HspB8, or by the Bradford method [44] for HspB7.

### 4.2. Oligomeric Structure of ACDs

We analyzed the oligomeric structure of the ACDs by size-exclusion chromatography on a Superdex 75 10/300 GL column (GE Healthcare, Chicago, IL USA) equilibrated by buffer A (50 mM phosphate, pH 7.4, containing 150 mM NaCl and 2 mM DTT). The samples (25 μL) containing variable quantities (16–150 μg or 25–600 μM per monomer) of protein were loaded onto the column and eluted at a rate of 0.75 mL/min. We recorded the elution profiles at 214 nm and calibrated the column with the following standards: RNase (13.7 kDa), carbonic anhydrase (29.0 kDa), ovalbumin (43.0 kDa) and conalbumin (75.0 kDa).

### 4.3. SEC–MALS

We applied size-exclusion chromatography with multiangle light scattering (SEC–MALS) detection to determine the absolute Mw values for B1ACD and B8ACD. The SEC–MALS measurements were performed at the Shared-Access Equipment Centre “Industrial Biotechnology” of the Federal Research Center “Fundamentals of Biotechnology” of the Russian Academy of Sciences. We loaded the proteins (2 mg/mL, 90 µL) on a Superdex 200 Increase 10/300 column (GE Healthcare) at a 0.8 mL/min flow rate. The column was pre-equilibrated with filtered (0.1 μM) and degassed 20 mM Tris–HCl buffer (pH 7.5), containing 150 mM NaCl and 3 mM β-mercaptoethanol. We assessed the elution profiles by using a combination of the sequentially connected UV–Vis Prostar 335 (Varian, Belrose, Australia) and miniDAWN detectors (Wyatt Technology, Santa Barbara, CA, USA) and processed the data in ASTRA 8.0 (Wyatt Technology, Santa Barbara, CA, USA) using a dn/dc equal to 0.185 and extinction coefficient A_280_^0.1%^ values equal to 0.87 and 0.73 for the B1ACD and B8ACD, respectively. These ACDs were selected for analysis because the other three ACDs had extremely low extinction coefficients (<0.2), which could have compromised the accuracy of their absolute Mw determination. In addition, we determined the apparent Mw values from the column calibration with standard proteins. We built the plots using Origin 9.0 (Originlab, Northampton, MA, USA).

### 4.4. DLS

We performed the DLS experiments at 25 °C on NanoZS (Malvern, Malvern, WR14 1XZ, UK) in buffer B (20 mM Tris–acetate, pH 7.6, 10 mM NaCl, 0.1 mM EDTA, 0.1 mM PMSF) and a protein concentration equal to 3.0 mg/mL. We repeated ten runs ten times at 25 s each and determined the particle diameters in the intensity mode.

### 4.5. Chemical Crosslinking

We mixed the ACDs (3 mg/mL) in 25 mM HEPES (pH: 7.5) with a water solution of glutaraldehyde (final concentration: 0.008% or 0.024%) and incubated them for 30 min at 30 °C. We stopped the reaction with the addition of the SDS sample buffer, loaded equal volumes and quantities of the proteins onto 15% polyacrylamide gel and ran them on the Tricine–SDS–PAGE [43].

### 4.6. Incorporation of ACDs into Oligomers of Full-Length Small Heat Shock Proteins

We used two methods to assess the incorporation of the ACDs into the oligomers formed by the full-length sHsps. In the first case, for analyzing the interaction of ACDs with sHsps that form large oligomers, we used size-exclusion chromatography. In this case, we mixed the full-length sHsps (~19–20 μM per monomer) with different ACDs (38–110 μM per monomer) in buffer B. After 30 min of incubation at 42 °C, we loaded 100 μL of the isolated proteins or their pair mixtures onto a Superdex 200 Increase 30/100 column (GE Healthcare, Chicago, IL, USA), equilibrated by buffer A, and eluted them at a rate of 0.75 mL/min. We recorded the elution profiles at 214 nm, collected 0.4 mL of the fractions and analyzed their protein compositions by SDS gel electrophoresis [45]. The column was calibrated by the following standards: RNase (13.7 kDa), carbonic anhydrase (29.0 kDa), ovalbumin (43.0 kDa), conalbumin (75.0 kDa), aldolase (158.0 kDa), ferritin (440.0 kDa) and thyroglobulin (660.0 kDa).

In the second case, to analyze the interactions of the ACDs with the sHsps that predominantly form small oligomers, we used native clear (stainless) gel electrophoresis that we ran on 12.5% polyacrylamide gels in homogeneous 80 mM glycine–Tris (pH: 8.6) [32]. We mixed the sHsps that tended to form small oligomers (3D mutant of HspB1, HspB6, HspB7 and HspB8) (0.10–0.12 mg/mL and 5–6 μM per monomer) with different ACDs (0.20–0.24 mg/mL and 20–24 μM per monomer) and incubated them for 30 min at 25 °C in buffer B. The samples containing the isolated ACDs and those containing the corresponding sHsps were treated identically. Afterwards, we subjected the individual proteins and their mixtures to electrophoresis. We considered the appearance of a new band possessing an electrophoretic mobility different from those of the isolated proteins and ACDs as an indication of the incorporation of the ACDs into the oligomers of the full-length sHsps. Immunoblotting supported this conclusion. After the electrophoresis, we washed the gels 5 times in 25 mM Tris, 192 mM glycine, 0.1% SDS, pH 8.3 and blotted the proteins on nitrocellulose. We blocked the nitrocellulose with 5% nonfat dry milk in TBST and incubated it with mouse monoclonal anti-HspB6 antibodies (HyTest, Turku, Finland). After washing, we incubated the nitrocellulose with secondary anti-mouse goat antibodies conjugated with peroxidase and visualized the protein bands by enhanced chemiluminescence (Invitrogen, Waltham, MA, USA). This approach cannot be applied to the sHsps that predominantly form large oligomers, such as wild-type HspB1 or HspB5, since due to their high molecular weights, these proteins have only low electrophoretic mobilities and form diffuse bands on the gel top.

## Supplementary Materials

The following supporting information can be downloaded at: https://www.mdpi.com/article/10.3390/ijms24021085/s1.

## Abbreviations

ACD: α-crystallin domain; DLS: dynamic light scattering; DTT: dithiothreitol; PMSF: phenylmethane sulfonyl fluoride; sHsps: small heat shock proteins; TBST: Tris-buffer saline containing Triton.
