# Supplementary material for: α-Crystallin Domains of Five Human Small Heat Shock Proteins (sHsps) Differ in Dimer Stabilities and Ability to Incorporate Themselves into Oligomers of Full-Length sHsps
Source: Int J Mol Sci. 2023 Jan 6;24(2):1085. doi: 10.3390/ijms24021085 (PMC9860685; doi:10.3390/ijms24021085)
Supplement: Supplementary file 1 [file ijms-24-01085-s001.zip › ijms-2055539-Supplementary Material Figure S1.pdf]

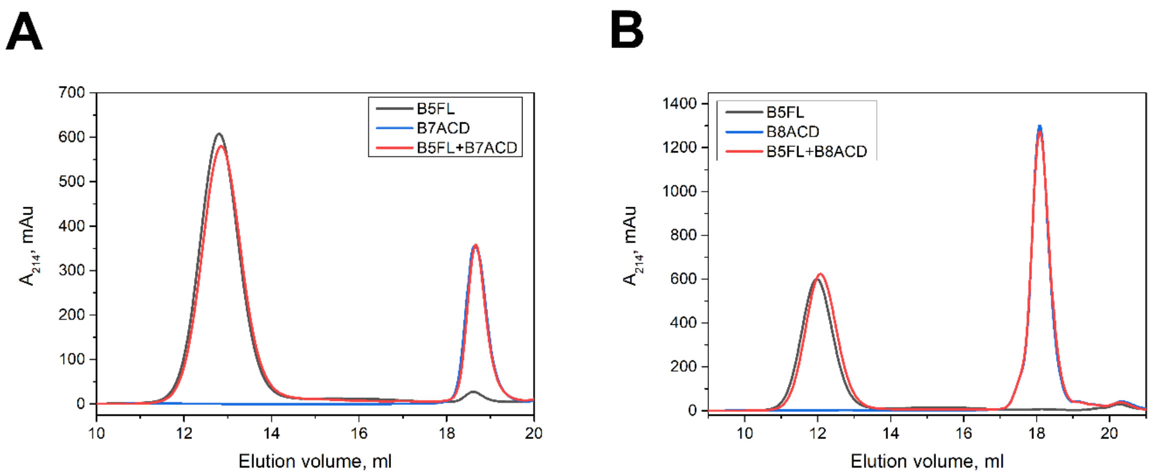

Supplementary Material Figure S1. Interaction of B7ACD (A) and B8ACD (B) with HspB5. Size-exclusion chromatography of HspB5 (black lines), B7ACD and B8ACD (blue lines) and the equimolar mixture of HspB5 and B7ACD or B8ACD (red lines). Representative results of no less than three experiments are presented.
