# Supplementary material for: α-Crystallin Domains of Five Human Small Heat Shock Proteins (sHsps) Differ in Dimer Stabilities and Ability to Incorporate Themselves into Oligomers of Full-Length sHsps
Source: Int J Mol Sci. 2023 Jan 6;24(2):1085. doi: 10.3390/ijms24021085 (PMC9860685; doi:10.3390/ijms24021085)
Supplement: Supplementary file 1 [file ijms-24-01085-s001.zip › ijms-2055539-Supplementary Material Figure S3.pdf]

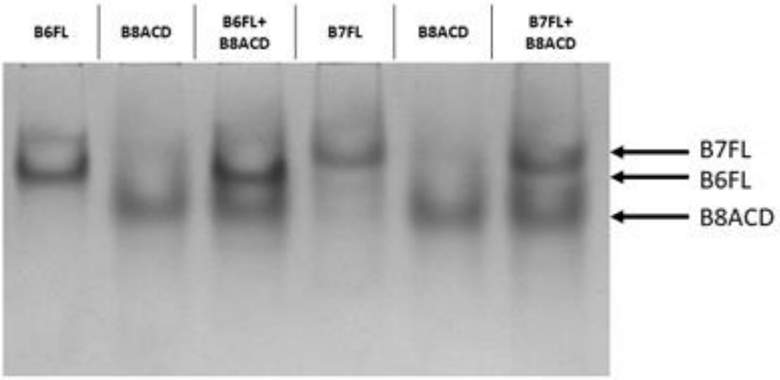

Supplementary Material Figure S3. Interaction of full-length HspB6 (B6FL) and full-length HspB7 (B7FL) with B8ACD studied by native PAGE. Positions of full-length proteins and crystallin domains are marked by arrows. Representative results of no less than five experiments are presented.
