# Supplementary material for: α-Crystallin Domains of Five Human Small Heat Shock Proteins (sHsps) Differ in Dimer Stabilities and Ability to Incorporate Themselves into Oligomers of Full-Length sHsps
Source: Int J Mol Sci. 2023 Jan 6;24(2):1085. doi: 10.3390/ijms24021085 (PMC9860685; doi:10.3390/ijms24021085)
Supplement: Supplementary file 1 [file ijms-24-01085-s001.zip › ijms-2055539-Supplementary Material Figure S4.pdf]

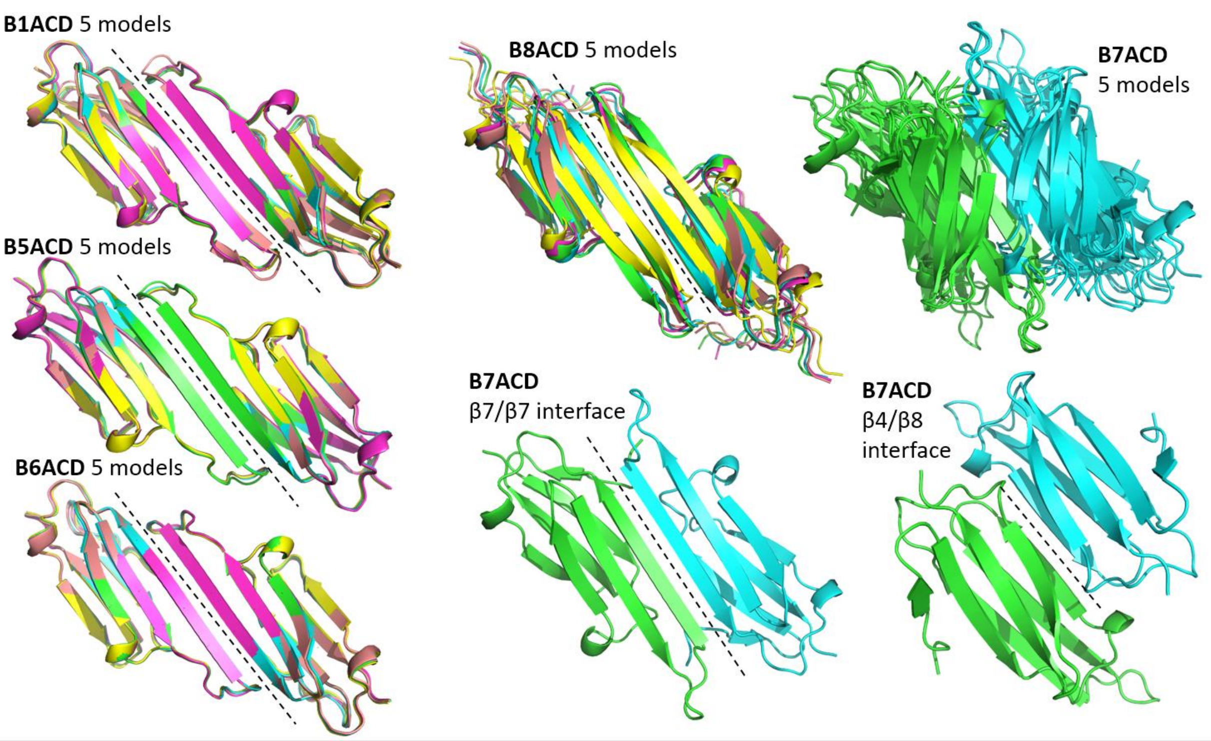

Supplementary Material Figure S4. Analysis of the preservation of the canonical ACD dimer interfaces in selected sHSP members as predicted by Alphafold2 (Mirdita M et al., Nature Methods 2022 679) https://doi.org/10.1038/s41592-022-01488-1). Five models were calculated for B1ACD, B5ACD, B6ACD, B7ACD and B8ACD in a multimer mode using two ACD chains for each protein. The resulting AMBER-relaxed models were then superimposed. Note that while Ca RMSD did not exceed 1 Å for B1ACD, B5ACD and B6ACD among the five models calculated, suggesting the preservation of the canonical dimerization interface, the superposition of B8ACD models showed slight variation in the models and interfaces. In the case of B7ACD, no stable solution could be found, and subunits contacted using the opposite interfaces, implying difficulties in assembling the canonical ACD dimer. The models are shown by ribbon diagrams using different colors. The dashed lines indicate the dimeric interfaces.
