# Supplementary material for: α-Crystallin Domains of Five Human Small Heat Shock Proteins (sHsps) Differ in Dimer Stabilities and Ability to Incorporate Themselves into Oligomers of Full-Length sHsps
Source: Int J Mol Sci. 2023 Jan 6;24(2):1085. doi: 10.3390/ijms24021085 (PMC9860685; doi:10.3390/ijms24021085)
Supplement: Supplementary file 1 [file ijms-24-01085-s001.zip › ijms-2055539-Supplementary Material Table S1.pdf]

Supplementary Material Table S1. Primers used for obtaining constructs coding for α-crystallin domains

Primer	Sequence 5`->3`
HspB1-ACD-84-fw-NdeI	agatata**catatg**ggagtatcagaaatacgtcacacagctgaccgctgg
HspB1-ACD-170-rev-XhoI	gatc**ctcgag**ttagggcatgggggcctccacgg
HspB5-ACD-64-fw-NdeI	gatc**catatg**ggactctcagagatgcgcct
HspB5-ACD-150-rev-XhoI	gatc**ctcgag**ttatttccttggtccattcacagt
HspB6-ACD-64-fw-NdeI	gatc**catatg**gtcgcccaggtgccgacggac
HspB6-ACD-149-rev-XhoI	gatc**ctcgag**tta cgctggtgcggcctggatgga
HspB7-ACD-71-fw-NdeI	gatc**catatg**gggcaggcaacatcaagaccta
HspB7-ACD-154-rev-XhoI	gatc**ctcgag**ttagtgacgccgtgcccggatagt
HspB8-ACD-85-fw-NdeI	agatata**catatg**ggaagaacaccaccaccatttcccggagaaccctgg
HspB8-ACD-171-rev-XhoI	gatc**ctcgag**ttagacctggggagcttcgatgatcagc

Bold case letters highlight restriction sites
