# Supplementary material for: α-Crystallin Domains of Five Human Small Heat Shock Proteins (sHsps) Differ in Dimer Stabilities and Ability to Incorporate Themselves into Oligomers of Full-Length sHsps
Source: Int J Mol Sci. 2023 Jan 6;24(2):1085. doi: 10.3390/ijms24021085 (PMC9860685; doi:10.3390/ijms24021085)
Supplement: Supplementary file 1 [file ijms-24-01085-s001.zip › ijms-2055539-Supplementary Material Table S2.pdf]

Supplementary Material Table S2. Some properties of five α-crystallin domains analyzed in this work

	**Total number of residues**	**Mol. Mass, Da**	**pI**
**B1ACD (84-170)**	**87**	**9703**	**5.46**
**B5ACD (64-150)**	**87**	**9953**	**6.21**
**B6ACD (64-149)**	**86**	**9390**	**5.73**
**B7ACD (71-154)**	**84**	**9156**	**4.97**
**B8ACD (85-171)**	**87**	**9569**	**5.65**
